# Pathomechanisms of Immunological Disturbances in β-Thalassemia

**DOI:** 10.3390/ijms22189677

**Published:** 2021-09-07

**Authors:** Anna Gluba-Brzózka, Beata Franczyk, Magdalena Rysz-Górzyńska, Robert Rokicki, Małgorzata Koziarska-Rościszewska, Jacek Rysz

**Affiliations:** 1 Department of Nephrology, Hypertension and Family Medicine, Medical University of Lodz, 90-549 Lodz, Poland; bfranczyk-skora@wp.pl (B.F.); malgorzata.koziarska-rosciszewska@umed.lodz.pl (M.K.-R.); jacek.rysz@umed.lodz.pl (J.R.); 2 Department of Ophthalmology and Visual Rehabilitation, Medical University of Lodz, 90-549 Lodz, Poland; mrs-89@o2.pl; 3 Clinic of Hand Surgery, Medical University of Lodz, 90-549 Lodz, Poland; robert.rokicki@umed.lodz.pl

**Keywords:** β-thalassemia, anemia, immunological disturbances

## Abstract

Thalassemia, a chronic disease with chronic anemia, is caused by mutations in the β-globin gene, leading to reduced levels or complete deficiency of β-globin chain synthesis. Patients with β-thalassemia display variable clinical severity which ranges from asymptomatic features to severe transfusion-dependent anemia and complications in multiple organs. They not only are at increased risk of blood-borne infections resulting from multiple transfusions, but they also show enhanced susceptibility to infections as a consequence of coexistent immune deficiency. Enhanced susceptibility to infections in β-thalassemia patients is associated with the interplay of several complex biological processes. β-thalassemia-related abnormalities of the innate immune system include decreased levels of complement, properdin, and lysozyme, reduced absorption and phagocytic ability of polymorphonuclear neutrophils, disturbed chemotaxis, and altered intracellular metabolism processes. According to available literature data, immunological abnormalities observed in patients with thalassemia can be caused by both the disease itself as well as therapies. The most important factors promoting such alterations involve iron overload, phenotypical and functional abnormalities of immune system cells resulting from chronic inflammation oxidative stress, multiple blood transfusion, iron chelation therapy, and splenectomy. Unravelling the mechanisms underlying immune deficiency in β-thalassemia patients may enable the designing of appropriate therapies for this group of patients.

## 1. Introduction

Congenital hemolytic anemias (CHAs) comprise heterogeneous hereditary disorders associated with decreased life span and premature removal of the erythrocytes from the circulation [1]. One of them, β-thalassemia, a chronic disease with chronic anemia, is caused by mutations in the β-globin gene, leading to reduced levels or complete deficiency of β-globin chain synthesis and consequent ineffective functions of red blood cells [2,3,4]. The imbalance in the relative quantity of α-globin and β-globin chains and the formation of harmful reactive oxygen species (ROS) in erythroid progenitors leads to early apoptosis of maturing nucleated erythroid cells with hematopoietic expansion and subsequent chronic hemolytic anemia with significant reticulocytosis, severe anemia, and an array of secondary pathophysiologic mechanisms [5,6]. β-thalassemia is an autosomal recessive disease [7]. Currently, over 100 mutations with the hemoglobin gene have been identified in individuals with β-thalassemia [8]. According to estimations, 59–70,000 patients are suffering from the major form of this genetic anomaly worldwide. Patients with β-thalassemia display variable clinical severity which ranges from asymptomatic features to severe transfusion-dependent anemia and complications in multiple organs [9]. Chronic hemolytic anemia is severe and fatal in most homozygous cases unless repetitive blood transfusions are initiated early [7]. Moreover, infectious complications and immune aberrations affecting both innate and adaptive immunity are considered to be an important cause of morbidity and mortality in patients with β-thalassemia (the second most common cause of death in these patients) [10,11]. Patients with thalassemia are not only at increased risk of blood-borne infections resulting from multiple transfusions, but they also show enhanced susceptibility to infections as a consequence of coexistent immune deficiency. Enhanced susceptibility to infections in β-thalassemia patients is associated with the interplay of several complex biological processes and was demonstrated to be also affected by external factors, such as blood transfusion, iron chelation therapy, splenectomy, as well as vaccination status [12]. The presence of β-thalassemia is also associated with several abnormalities of the innate immune system observed from early childhood. They include: decreased levels of complement, properdin and lysozyme, reduced absorption and phagocytic ability of polymorphonuclear neutrophils, disturbed chemotaxis, and altered intracellular metabolism processes (higher activity of peroxidase, chloroacetate esterase, acid, and alkaline phosphatase, and a lower glycogen content) have been described in the literature [8,10]. The disruption of monocyte-macrophage system chemotaxis and phagocytosis could be induced by multiple blood transfusions as a consequence of chronic immune stimulation by foreign proteins [8]. The first and most common cause of death in thalassemia patients is cardiac failure, while hepatic disease is the third most frequent cause [7,13]. The standard treatment that is now available for β- thalassemia patients involves a lifelong hemotransfusion therapy in order to preserve normal levels of hemoglobin and to suppress enhanced but ineffective erythropoiesis [8]. Unfortunately, multiple transfusions unavoidably result in the development of alloimmunization and the accumulation of iron in tissues leading to enhanced risk for transmitted infections [8]. Repeated transfusions can also lead to severe iron overload, and in consequence, to progressive organ failure [2,14]. 

## 2. Molecular Pathogenesis of Beta-Thalassemia

The main pathophysiological mechanism underlying β-thalassemia is associated with unbalanced production of α-globin and β-globin chains in which α-globin chains seem to be in excess [15,16]. Since α-globin chains, in contrast to β-globin chains, cannot form stable tetramers, free excessive α-globin chains form insoluble aggregates that tend to precipitate within the developing erythroid cell, thus resulting in the stimulation of apoptosis in the developing erythroid precursor (ineffective erythropoiesis) [17]. Such precipitated α-globin chains are present in the cytoplasm and the nucleus, and the majority in polychromatic erythroblasts. Moreover, the accumulation of free α-globin chains initiates the generation of reactive oxygen species in the small percentage of erythroid cells which progress to maturation, enhancing the oxidative stress and leading in consequence to red blood cells (RBC) membrane damage followed by amplified hemolysis [15,18]. In thalassemia, free α- and β-globin chains are susceptible to the oxidation of hemichromes, which are the form of low-spin methemoglobin that cannot be reduced back to hemoglobin, instead of methemoglobin [19]. Such irreversible modification of globin chains enables the hemichrome iron to generate reactive oxygen species [20]. In β-thalassemic patients, the presence of anemia is associated with the ineffective erythropoiesis of the developing erythroid precursor cells accompanied by augmented hemolysis of the mature RBC, and it leads to a feedback loop involving enhanced expansion of erythroid progenitors and hastened erythroid differentiation [19]. The presence of significantly amplified erythropoiesis in beta-thalassemia has been confirmed by many studies [15,17,21]. In intermediate and severe beta-thalassemia cases, striking expansion of the erythroid mass is associated with organ enlargement as well as bone deformity and fragility [22,23]. Moreover, the incomplete loss of nuclear membrane and incidence of intranuclear aggregates of α-globin chains within erythroid nuclei have been observed in bone marrow erythroblasts of homozygous patients. In β-thalassemia major, the occurrence of apoptosis erythroid precursors at the polychromatophilic normoblast stage (α-globin chain aggregation) was reported [17].

Moreover, the combination of severe anemia resulting from ineffective erythropoiesis with substantial tissue hypoxia stimulates the production of erythropoietin (EPO) to a greater extent compared with normal controls [24]. The results of some studies imply that elevated levels of EPO are responsible for the expansion of the erythroid mass, mainly in the bone marrow, liver, and spleen, as well as at extramedullary sites [15].

## 3. Pathogenesis of Immune Defects in Thalassemia

### 3.1. Phenotypical and Functional Abnormalities

A wide spectrum of immune defects has been observed in β-thalassemic patients [7,25]. The presence of a compromised innate immune system contributes to poor outcomes following infection. These quantitative and functional abnormalities concern several components of the immune response. Altered cytokine profile of innate immunity as well as the presence of low-grade systemic inflammatory status reflected by increased total leukocyte, neutrophil, and lymphocyte counts have been demonstrated in this group of patients [11]. The abnormal neutrophil effector function plays a fundamental role in infection susceptibility in these patients. Moreover, in these patients, alterations in T-lymphocyte subsets including increased level and activity of suppressor T cells (CD8), diminished count and activity of helper T cells (CD4) resulting in reduced CD4/CD8 ratios, decreased T cells’ proliferative capacity and defective activity of Natural Killer (NK) cells [7]. Furthermore, the amount of B lymphocytes is higher and their activation is enhanced, while differentiation is impaired [7]. Elevated levels of immunoglobulins IgG, IgM, and IgA indicate compromised immunoglobulin secretion. Defective chemotaxis and phagocytosis have also been described in thalassemic patients. Finally, reduced opsonization and granulocyte phagocytosis as well as suppressed functioning of the complement system, mirrored by decreased levels of C3 and C4, has also been reported [7,10,26,27,28]. 

### 3.2. Polymorphonuclear Neutrophils (PMN)/Granulocytes (Neutrophils, Eosinophilic Cells, Basophilic Cells)

Mechanisms involved in the dysfunctional innate immune system comprise defects in neutrophil migration, phagocytosis, and the activation of pathogen-specific immune responses [12]. The exact reasons for the failure of neutrophils to mount appropriate responses remain indefinite. Neutrophils of thalassemic patients display considerably decreased functional activity compared to those isolated from healthy controls [29]. Kyriakou et al. [30] demonstrated the overexpression of CD11b, CD18, CD35, CD44, and CD67 on the surface of neutrophils. In turn, Buttari et al. [9] reported in thalassemic patients CD16+ neutrophils phenotype and function abnormalities which varied according to the clinical form of the disease and to the neutrophil subset (CD16bright and CD16dim). The aforementioned aberrations included altered surface expression of the innate immune receptor CD45, Toll-like receptor 4, and CD32, as well as elevated levels of membrane lipid peroxidation, specifically in patients with a more severe form of the disease. Moreover, neutrophils of thalassemic patients were found to show high expression of apoptotic markers—caspases—in some but not all studies [11,25,31,32]. Neutrophils are unquestionably chief effectors of acute inflammation; however, the results of some studies have indicated that they can also contribute to chronic inflammatory conditions and adaptive immune responses [33]. Recently, it was found that some extravasated neutrophils might re-enter circulation, thus leading to the dissemination of inflammation to other organs and subsequent tissue injury [33]. Chemokines such as CXCL1 and CXCL2, displaying the affinity to CXCR1 and CXCR2 receptors, respectively, coordinate the process of neutrophils’ migration towards the site of infection [12]. CXCR2 has been confirmed to be a vital component of the neutrophil chemotactic response [33]. Siwaponanan et al. [12] demonstrated with the use of an animal model of thalassemia a serious impairment in the CXCR2–CXCL2 axis, resulting in considerably decreased CXCR2 expression in neutrophil cells upon exposure to *Streptococcus pneumoniae*. The predisposition of patients with this disease to life-threatening bacterial infections could be related to defective neutrophils; however, molecular and cellular mechanisms responsible for compromised neutrophil function have not been fully understood [12]. It seems that inability to respond to chemokines may lead to compromised neutrophil migration and diminished resistance to infection [34]. In patients with β-thalassemia, impaired neutrophil phagocytosis was observed [7,10]. The same observation was made in Hbbth3/+ neutrophils in the animal model [12]. Moreover, such neutrophils showed a significant reduction in CD11b (one of the CR3 subunits) expression which resulted in their impaired phagocytic activity following stimulation with *Staphylococcus aureus* [12]. The results of animal studies suggested that deficient innate immunity against bacteria, especially the phagocyte system, could be responsible for low immunity in β-thalassemia [35]. Ren et al. [36] found that CR3 deficiency is associated with life-threatening infections, while the diminished expression of CD11b may contribute to increased susceptibility to infections in an animal model of β-thalassemia. The process of recognition and binding of pathogens requires the participation of neutrophil surface receptors, including pattern-recognition receptors, such as Toll-like receptors, Fcγ receptors, and the CRs [33,37]. CR3, which is highly expressed neutrophils, monocytes, macrophages, and NK cells, has been demonstrated to play a vital role in leukocyte adhesion, migration, and phagocytosis [38]. Apart from chemotaxis, phagocytosis, the antibacterial effector actions of the phagocytes, involves the generation of a respiratory burst of ROS [39,40]. To fight with microbes, polymorphonuclear neutrophils (PMN), in response to bacterial components, and phorbol-myristate-acetate (PMA) generate a burst of reactive oxygen species [31]. The generation of ROS in the course of respiratory burst requires the activity of nicotinamide adenine dinucleotide phosphate (NADPH) oxidase [41]. Generated superoxide anion (O_2_^−^) is transformed in the process mediated by superoxide dismutase into oxygen and hydrogen peroxide (H_2_O_2_), which is a part of a powerful germ-killing system of the PMN. The NADPH complex contains cytosolic (such as p47phox, p67phox, p40phox, and the small G-proteins, Rac2 and Cdc42) and membrane components (gp91phox and p22phox), which interact following activation. The results of animal studies indicated a considerable decline in p40phox and p47phox components in blood and spleen as well as disturbed upregulation of p22phox, p91phox, p40phox, and p67phox expression in splenocytes following *S. pneumoniae* infection [31]. Moreover, Amer et al. [42,43] found that ROS content in polymorphonuclear neutrophils in thalassemia is higher compared to normal PMN, and these neutrophils were less capable of responding to PMA (phorbol 12-myristate 13-acetate) activation, which may imply their impaired bactericidal activity. Cantinieaux et al. [44] suggested that elevated ROS in PMN and compromised ability of PMN to respond to PMA were associated with increased levels of iron and hemin in the plasma of thalassemic patients. The presence of additional oxidative stress was found to destabilize the secondary lysosomes of phagocytic cells leading in consequence to loss of their protective function [8]. Amer et al. [43] also revealed decreased content of reduced glutathione and enhanced membrane lipid peroxidation in RBC of thalassemic patients. On the basis of obtained results, they suggested that decreased resistance to bacterial infections in β-thalassemia syndromes stemmed from chronic oxidative stress and the consequent diminished ability of PMN to respond via respiratory burst to bacterial components [31]. This raises the question of whether the administration of antioxidants could enhance defense mechanisms against the severe complications of recurrent infections in β-thalassemia. Preliminary studies assessing the effects of treatment of thalassemic PMN with antioxidant (*N*-acetylcysteine) indicated a reduced basal ROS level and a higher ability of polymorphonuclear neutrophils to respond to PMA [31]. Moreover, the analysis of thalassemic splenic neutrophils revealed a high count of immature band cells, which could provide an explanation for less efficient phagocytosis and ROS production [45]. Neutrophils isolated from patients with β-thalassemia were found to be hyposegmented and displayed reduced expression of ets-family transcription factor PU.1, which demonstrates an aberrant or arrested state of neutrophil maturation [46,47]. According to studies, the expression of PU.1—a key myeloid regulator—is associated with maturation arrest and neutrophil functional defects observed in an animal model of thalassemia [48]. PU.1 was found to synchronize the expression of myeloid genes responsible for numerous activities, including terminal maturation (G-CSFR), phagocytosis (CD11b/CD18), ROS formation (NADPH oxidase), as well as the promotion of apoptosis via direct transactivation of apoptosis-related genes (especially tumor necrosis factor-related apoptosis-inducing ligand) [49]. Siwaponanan et al. [12] confirmed a considerable reduction in the expression of PU.1 in neutrophils isolated from Hbbth3/+ mice with aberrant and/or arrested state of neutrophil maturation. In turn, in humans, significantly reduced or absent PU.1 expression was found in a condition characterized by maturation arrest of myeloid cells—acute myeloid leukemia [47,50,51]. This mechanism could also be responsible for the enhanced survival of dysregulated neutrophils. The evidence from the study of blood neutrophils isolated from HbE/β-thalassemia patients confirmed significantly diminished PU.1 expression in peripheral blood neutrophils compared with healthy controls [12]. 

### 3.3. Lymphocytes (T Cell, B Cell, NK Cells)

Some studies of thalassemic patients have reported the presence of several phenotypically unique T-lymphocyte subsets with unique functions [52]. Thalassemic patients were found to have elevated levels of suppressor T cells, helper T cells, natural killer cells, and B cells, especially CD3+/CD4+, CD3+/CD8+, CD3-/CD16/56+, and CD3-/CD19+ [25,53,54,55]. According to studies, the increase in the ratio of T regulatory (CD4+/CD25+/Foxp3+) lymphocyte subset, responsible for the negative control of immune responses against foreign and one’s own antigens as well as the inhibition of immune cells (B and T cells) and antigen-presenting dendritic cells, is the most noticeable characteristic of altered lymphocytic subpopulations in TM (thalassemia major) patients [56,57,58]. The increased levels of activated T cells and serum neopterin may imply chronic stimulation of the immune system in thalassemia patients [59]. Moreover, the activity of natural killer cells (NKCs) was found to be reduced in this group of patients [60]. Gharagozloo et al. [59] demonstrated considerably elevated absolute lymphocyte counts in patients with thalassemia compared with the control group, which probably resulted from a persistent antigenic challenge from blood transfusions. A higher number of activated T cells was accompanied by an increased concentration of neopterin, a proinflammatory mediator synthesized by activated macrophages, which implies the presence of chronic stimulation of the immune system in this group of patients. However, in their study, the levels of T-cell proliferation and interleukin 2 (IL-2) as well as IL-4 and interferon-gamma (IFN-gamma) production were considerably suppressed in patients. Decreased IFN-gamma and IL-2 levels in subjects with elevated ferritin levels indicate, according to authors, the immunosuppressive effect of iron overload in beta-thalassemia patients [59]. Therefore, the double-faced immune response observed in thalassemic patients could be the result of continuous immune stimulation as well as multiple blood transfusions. Cellular components seem to be impoverished in thalassemic patients; however, humoral determinants of innate immunity appear amplified in them. Oxidative stress has been suggested to be involved in this phenomenon [61]. 

B lymphocytes play an important role in the production of autoantibodies and alloantibodies against transfused red blood cells, and therefore, they are of high importance for the functioning of humoral immunity in thalassemic patients. The greater proportion of B cells, especially those with regulatory phenotype, expressing CD19, CD38, and CD24, but no difference in ratios of T cell subpopulation has been demonstrated in this group of patients [52]. Kyriakou et al. [30] reported the overexpression of CD38 and CD69 on the surface of lymphocytes in patients with thalassemia. In turn, Ghaffari et al. [62] observed considerably increased levels of IgA immunoglobin, but no difference in IgG, IgM, and IgE levels or complement components of C3 and C4. The suppression of T-cell immune response was suggested to be associated with an inflammatory status mirrored by elevated levels of IL-17 and transforming growth factor β (TGF-β), but not IL-21 [2]. Similar results were obtained by Balouchi et al. [9] who implied that despite the fact that T cells showed a stimulated phenotype in thalassemic patients, their activity was inhibited. Another study indicated considerably decreased production of IL-2, interferon gamma (IFN-γ), and IL-4 by activated lymphocytes from patients with β-thalassemia compared to the normal group [59]. 

In patients with thalassemia, the phenomenon of immunosenescence has been reported. This state is characterized by premature aging of lymphocytes [63]. Apart from oxidative stress, prolonged activation of T cells in the state of chronic infections has also been suggested as a factor increasing the rate of T-cell replicative senescence resulting in the increase in the count of T cells with altered function and proliferation inability [61]. Reduced expression of co-stimulatory molecule CD28 enables the phenotypical distinguishing between senescent lymphocytes with reduced proliferative capacity and normal ones [25,61]. This molecule plays a critical role in T-cell activation, proliferation, and survival [61,64]. It has been found that CD8+ T cells lacking the expression of CD28 show some atypical features, including diminished T-cell receptor diversity, defective proliferation during antigen stimulation, and a suppressive effect on CD4 T-cell activation [65,66]. Gharagozloo et al. [63] reported that the percentages of CD8+/CD28- and CD3+/CD95+ T lymphocytes were much higher in thalassemia patients, representing senescent T lymphocyte phenotypes. T CD8+28- cells decrease the immune response to pathogens and new infections [65]. They also participate in adaptive immunity since they convert dendritic cells (DCs) to tolerogen via the lowering of co-stimulatory molecule expression on DCs [67]. Moreover, Gharagozloo et al. [63] found greater resistance of lymphocytes from beta-thalassemia patients to spontaneous apoptosis compared to normal lymphocytes as well as reduced telomerase activity of activated T cells. In turn, Hsu et al. [68] observed the association between the presence of increased T cell counts displaying a higher expression of CD 95, Fas apoptotic receptor, and senescent nature of the lymphocyte population. The results of studies suggest that in β-thalassemia major patients, the augmentation of senescence T cells, which impairs the functioning of the immune system, could be one of the reasons for greater susceptibility of infections [61].

Moreover, the expansion of immature myeloid cells in the spleen was found to be as high as in the bone marrow. Earlier studies have demonstrated that such expansion in the spleen, liver, lung, and other lymphoid organs is observed in pathological conditions, including autoimmunity, cancers, and cystic fibrosis [51,69,70,71]. It has been suggested that the presence of an expanded number of myeloid-derived suppressor cells (MDSCs) may be involved in progressive organ damage in β-thalassemia [12]. MDSCs are frequently found in inflammatory settings in which they suppress T-cell and NK functions [51]. 

## 4. The Causes Leading to Changes in the Immune System

According to available literature data, immunological abnormalities observed in patients with thalassemia can be caused by both the disease itself as well as therapies [8]. The most important factors promoting thalassemia-induced alterations of the immune system involve iron overload and the above-mentioned phenotypical and functional abnormalities resulting from chronic inflammation and oxidative stress. 

### 4.1. Disease-Related Factor

β-thalassemia itself results in a constant immune stimulation [8]. Reduced synthesis of β-globin chains translates into an excess amount of α-chains which precipitate in the precursors of erythrocytes, leading in consequence to structural changes of the cell membrane. Wanachiwanawin et al. [72] demonstrated that the presence of β-thalassemic abnormal erythrocytes resulting from excessive unbound α-globin stimulates a continuous activation of monocytes, which are responsible for immune clearance. This state is aggravated by multiple blood transfusions which promote autoimmune hemolysis, alterations of T-and B-lymphocytes, as well as the compromised functions of monocytes and macrophages [8,73].

Furthermore, the development of iron overload is associated with enhanced intestinal iron absorption (compensatory reaction induced by chronic anemia) related to ineffective erythropoiesis resulting from premature intramedullary death of erythrocytes as well as enhanced peripheral destruction of red cells; however, it could be also secondary to regular transfusions [6,8]. Iron overload in thalassemia syndromes was also found to be associated with growth differentiation factor 15 (GDF15) overexpression related to an expanded erythroid compartment and the inhibition of hepcidin expression [74]. According to studies, a higher demand for iron, related to anemia, iron deficiency, and hypoxia, results in diminished expression of hepcidin, leading to the increase in transporter ferroportin (FPN) levels and a corresponding elevation in circulating iron [75]. It was also found that hepcidin–ferroportin interaction may be of key importance in the pathophysiology of hereditary hemochromatosis and the anemia of inflammation. The presence of inflammation induces hepcidin, causing the distinctive decrease in blood iron (hypoferremia of inflammation), which is believed to enhance host resistance to microbial infection [76]. Moreover, erythroferrone (ERFE), the main erythroid regulator of hepcidin, is one of the key players of iron overload in thalassemia syndromes [77]. Increased ERFE levels (resulting from erythropoietin-stimulated synthesis) was found to inhibit hepcidin synthesis, thus mobilizing cellular iron stores for use in heme and hemoglobin synthesis [77]. In case of ineffective erythropoiesis, abnormal overproduction of ERFE suppresses hepcidin and leads to iron overload, even in non-transfused patients.

Numerous studies have indicated that iron overload is the main precipitating factor of immune deficiency in β-thalassemia [7]. Indeed, immune system abnormalities associated with conditions involving augmented iron load (thalassemia, hemochromatosis) are characterized by diminished phagocytosis by the monocyte–macrophage system, changes in T-lymphocyte subsets (reflected by the increase in CD8 and the suppression of CD4), compromised immunoglobulin secretion, as well as hampering of the complement system function [7,78]. This factor is believed to be the vital contributor to immune deficiency in β-thalassemia. Not only iron, but also protein compounds comprising iron possess immunoregulatory properties; thus, their increased levels may have an adverse effect on immune balance [8]. It has been demonstrated both in vitro and in vivo that iron can be involved in the regulation of the expression of surface markers of T lymphocytes, thus modulating the expansion of different subpopulations of T cells. Gharagozloo et al. [59] observed that that the production of IFN-gamma and IL-2 was significantly lower in patients with high serum ferritin levels, which suggests the immunosuppressive effect of iron overload in beta-thalassemia patients. Reduced ability of lymphocytes to sequester excess iron in ferritin has been suggested as an explanation of the immune system abnormalities in iron-overloaded patients [7,78]. Both PMN and monocyte-macrophage systems participate in toxic iron purification as these cells have the ability to endocytose free iron and ferritin. Compromised phagocytosis activity of polymorphonuclear neutrophils resulting from iron overload is the consequence of the deleterious effect of ferritin-associated iron [79]. Moreover, high levels of plasma ferritin observed in thalassemic patients may stimulate the formation of anti-ferritin antibodies and subsequent enhanced production of circulating immune complexes [78]. Intense chelation therapy with desferrioxamine has been demonstrated to ameliorate some iron-induced symptoms which provide direct evidence confirming the role of iron overload in immune system disturbances [79]. Chelation therapy accompanies transfusions in order to prevent excessive iron load and its complications [7]. 

Apart from immunological abnormalities, iron overload can also lead to many complications, such as cardiac failure, siderosis, and endocrine abnormalities (hypothyroidism, hypogonadism, hypoparathyroidism, and diabetes mellitus), which can prove fatal in the course of the disease [7]. 

### 4.2. External Factors

Apart from the disease-related factors, enhanced susceptibility to infections in β-thalassemia patients can be associated with the presence of external causes, such as blood transfusion (resulting in iron overload and allogeneic stimulation), iron chelation therapy, and splenectomy [8,12].

Multiple blood transfusions are considered as chief pathogenetic mechanisms of immune abnormalities as they result in continuous alloantigen stimulation and subsequent disturbances of the immune balance [8,31,73,79]. Current transfusion therapy used in patients with thalassemia comprises a hyperhemotransfusion regime aiming to preserve the pretransfusion hemoglobin level of 100 g/L or higher [80]. It has been demonstrated that blood transfusions can lead to diminished delayed-type hypersensitivity and the consequent production of anti-idiotypic anti-clonotype antibodies [8]. Both allogenic mononuclear cells and soluble substances formed during blood components storage are involved in immunomodulation. Furthermore, soluble peptides of human leukocyte antigen (HLA) class I were found to contribute to immunomodulation. Multiple blood transfusions have been also demonstrated to be associated with autoimmune hemolysis [8,73]. Hemolysis occurring in the blood circulation of β-thalassemia patients increases heme concentrations, leading to enhanced oxidative stress resulting in infection susceptibility as well as cell death by stimulating the formation of free radical oxidative species (ROS) and eliciting inflammatory injuries [3,81,82,83]. Heme was confirmed to exert deleterious effects on the control of bacterial infections since it hinders phagocytosis and the migration of phagocytes [84,85]. Moreover, the activity of the enzyme responsible for heme catalysis, heme oxygenase 1 (HO-1), could intensify inflammation, bacterial growth, and survival in infected mice and human macrophage-like cells [86,87]. Santos et al. [88] confirmed enhanced expression of HO-1 in the liver, spleen, and kidney of β-thalassemic mice compared to wild-type mice. Moreover, higher levels of HO-1 were also found in EPO-mediated erythroid differentiation of fetal liver cells isolated from β-thalassemic fetuses. Based on the aforementioned results, the authors suggested that β-thalassemic erythroblasts contained improperly high levels of unbound heme subjected to continuous degradation by HO-1 [88]. The presence of higher baseline concentrations of HO-1 in β-thalassemia can be explained by increased levels of hemin (>50 μM) in serum from β-thalassemia patients compared to non-thalassemia individuals in whom its levels are undetectable [85]. Both hemin and the product of HO-1-mediated hemin degradation have been demonstrated to exert various effects on cellular immune functions [3]. For example, Zhong et al. [89] observed that hemin triggered the polarization of CD4 T cells to a subset of regulatory T cells (Treg). The treatment of normal human peripheral blood mononuclear cell (PBMC) with HO-1 activators, hemin, and cobalt protoporphyrin (CoPP) was found to be associated with reduced levels of IFN-γ and IL-10 following stimulation with *Burkholderia pseudomallei* (Bp) [3]. In addition, other studies demonstrated that the stimulation of HO-1 by CoPP compromised dendritic cell maturation, decreased CD4+ and CD8+ T-cell proliferation and IFN-γ production, as well as elevated IL-10 levels in response to LPS in vitro [90]. Moreover, HO-1 reduces the number of immune cells and promotes T cell apoptosis in the mice model [91]. The authors suggested that the overexpression of HO-1 contributed to sepsis-induced immunosuppression during late-phase sepsis via the stimulation of Th2 polarization and Treg function. Since the modulation of host immune responses by heme, hemin and HO-1 appears to enhance the susceptibility to bacterial infection, it may hold promise for future host-directed therapies. 

Chelation therapy accompanies transfusions in order to prevent excessive iron load and its complications [7]. However, some iron chelators which are still used in patients with thalassemia (desferrioxamine; DFO) were found to increase patients’ susceptibility to bacterial infections including the family Yersinia [8]. In healthy individuals, the Yersinia species display low pathogenicity; however, they become increasingly pathogenic in patients with iron overload treated with DFO [80].

Iron chelators used in thalassemic patients (mostly Desferrioxamine, Deferasirox, and Deferiprone) may also contribute to Zn deficiency [92]. This common feature of thalassemia is involved in the pathophysiology of immunodeficiency [93]. Similar to the above-mentioned iron, zinc is also an immunoregulator necessary for appropriate functioning of immune cells [8]. Decreased levels of zinc in thalassemic patients have been shown to induce changes in lymphocyte subpopulations and thymulin deficit [8]. Zn insufficiency has been suggested to be responsible for decreased cytokine production and proliferative activities observed in thalassemic patients, despite the presence of higher counts for the total and activated lymphocytes [59]. Tienboon et al. [94] revealed that nutritional intervention can improve proliferative responses of lymphocytes, and therefore, they suggested that nutritional deficiencies (including Zn) may be associated with the state of immune disturbances in thalassemic patients. 

Finally, splenectomy, which is a standard therapeutic intervention in β- thalassemia performed in order to remove the increased red blood cell consumption caused by hypersplenism, also causes a predisposition to infections and immune system modifications [7,95]. Due to the fact that the spleen is the reservoir of immunocompetent lymphocytes and the primary organ of immunological surveillance, its removal modifies the immune system. Splenectomy promotes not only quantitative lymphocyte changes without any functional impairment but also the exacerbation of the immunological effects of multiple transfusions as a result of decreased immune clearance [96]. The results of studies have demonstrated that patients who underwent splenectomy have significant absolute lymphocytosis and considerably higher neutrophil counts, but this procedure may not affect cytokine levels [2,59,97]. Moreover, splenectomized beta-thalassemia/Hb E were found to have a considerably decreased percentage of CD3(+) cells corresponding with the elevation in CD19(+) cells [54]. Splenectomy has also been suggested to boost the number of both CD4+ and CD8+ T cells in TM patients [59]. In turn, Al-Ofairi demonstrated reduced CD and 4+ lymphocyte counts of NKCs following splenectomy [98]. The production of cytokines (such as IL-2 and tumor necrosis factor α (TNF-α)) by activated immune cells appears to be enhanced in splenectomized TM patients [25,59,97]. This procedure also reduced the activity of immune cells and decreased IgA and IgM immunoglobulin levels as well as C3 level and the activity of the complement system [25,99]. Moreover, Ammar et al. [100] observed that splenectomized thalassemic patients displayed lower IgM memory B cells compared to non-splenectomized patients. These cells possess the ability to respond more rapidly to a re-encounter of the immunizing antigen with high affinity and exquisite specificity [101]. Moreover, spleen-resident IgM memory B cells harbor a distinct antigen recognition profile. It was found that poorly opsonized bacteria, such as encapsulated bacteria, are only cleared by the spleen in contrast to opsonized bacteria which can be effectively removed by macrophages in the spleen and liver [100]. In turn, Sari et al. [97] demonstrated lower activity of macrophages in splenectomized TM patients compared to non-splenectomized subjects. Following splenectomy, thalassemic patients may experience the transitional diminished activity of neutrophils [97,102]. Finally, Kurtoğllu et al. [103] found considerably lower CD35 expression on the erythrocytes in splenectomized β-TM patients compared to non-splenectomized patients. The main role of CD35 on erythrocytes is to remove circulating immune complexes (C4b- and C3b-coated particles) on which the complement system has been activated. However, it seems that the aforementioned immunological effects related to splenectomy may be transitional, and the basal levels are restored over time [25]. 

## 5. Conclusions

According to available data, functions of aberrant neutrophils translate into considerable risk for disease-associated morbidity and mortality, which is partially due to the fact that their actions are associated with pathological processes, including organ damage and wound healing [45,104,105]. The unravelling of mechanisms underlying immune deficiency in β-thalassemia patients may enable the designing of appropriate therapies for this group of patients. Currently, it seems that decreased expression of PU.1 expression may be partly responsible for aberrant neutrophil maturation and effector functions in β-thalassemia patients. Therefore, perhaps therapeutic strategies focusing on neutrophil replenishment and maturation may prove to be beneficial in the diminishing of comorbidities and improvement of life quality in this group of patients.

## Data Availability

Not applicable.
